# Supplementary material for: Cells Lacking PA200 Adapt to Mitochondrial Dysfunction by Enhancing Glycolysis via Distinct Opa1 Processing
Source: Int J Mol Sci. 2021 Feb 5;22(4):1629. doi: 10.3390/ijms22041629 (PMC7914502; doi:10.3390/ijms22041629)
Supplement: Supplementary file 1 [file ijms-22-01629-s001.zip › supplementary/Supplementary Figure Legends_final0204.docx]

**Supplementary Figure S1. Validation of stable downregulation of PA200**

Stable depletion of PA200 (shPA200) in SH-SY5Y neuroblastoma cell line was validated using quantitative real-time PCR (A) and Western-blot upon vehicle (DMSO) or 3 µM oligomycin treatment for 24h (B-C). Results of the quantitative real-time PCR are presented as mean ± SD (n=3). Statistical analysis was performed with unpaired student’s t-test using GraphPad Prism v. 8.2.1 software (**** indicates p < 0.0001).

(B-C) Western blot images show successful stable depletion of PA200 under normal conditions (B) and upon treatment with vehicle (DMSO) or 3 µM oligomycin (O) for 24h (C). Actin was used as an internal loading control. Images were taken by ChemiDoc imager and analyzed using Image Lab V 6.1 software.

**Supplementary Figure S2. Western blots of biological replicates showing mitochondrial complexes from total cell lysates and isolated mitochondria**

(A) Total cell lysates from control and shPA200 cells were analyzed by western blot. OXPHOS antibody cocktail was used to detect four subunits of the electron transport chain (ETC) complexes, CI-NDUFB8, CII-SDHB, CIII-UQCRC2, CIV-MTCOI, and one subunit for the ATP synthase, CV-ATP5A. Images were taken using a ChemiDoc Imager and the density of the protein was normalized to the mitochondrial Hsp 60.)

(B) Mitochondria fractionation from control and shPA200 cells were performed and analyzed by western blot. The OXPHOS antibody cocktail was used to detect four subunits of the electron transport chain (ETC) complexes, CI-NDUFB8, CII-SDHB, CIII-UQCRC2, CIV-MTCOI, and one subunit for the ATP synthase, CV-ATP5A. Images were taken using a ChemiDoc Imager and the density of the protein was normalized to the mitochondrial Hsp 60.

**Supplementary Figure S3. Hyperpolarized mitochondria and elevated ROS level following oligomycin treatment**

(A) Mitochondrial membrane potential of shPA200 and control cells was assessed using TMRE; cells were treated with either the vehicle (DMSO) or 3µM oligomycin for 24 hr. Puromycin was removed prior 24 hr performing every experiment. Data are presented as mean values ± SD for n >4 independent experiments. Data were analyzed using FlowJo_V10. Statistical analysis was performed using Anova. GraphPad Prism V8.2.1 was used for statistical analyses and significance was determined. (*** indicates p<0.0001).

(B) ROS level was determined by staining cells with 1µM of Carboxy-H2DCFDA for 30 min and was measured by flow cytometry. Results are displayed as mean ± SD of ten independent experiments and statistically significant differences were calculated using ANOVA test (*p< 0.05).

**Supplementary Figure S4. Whole western-blots of mitochondrial fission and fusion proteins for figure 8A**

PA200-deficient and corresponding control cells were treated with vehicle (DMSO) or mitochondrial inhibitors at the following final concentrations; 3 µM oligomycin (O), 10 µM rotenone (R), and 100 nM antimycin A (A) for 24h.

(A-B) Western blot image of mitochondrial fission proteins; Drp1, Fis1 (A), and Mff (B).

(C) Western blot image of mitochondrial fusion proteins; Mfn1 and Mfn2. Actin was used as an internal loading control. Images were taken by ChemiDoc imager and analyzed using Image Lab V 6.1 software.

**Supplementary Figure S5. Whole western-blots of mitochondrial fusion protein OPA1, OMA1 and YME1L for figure 8B-D**

PA200-deficient and corresponding control cells were treated with vehicle (DMSO) or mitochondrial inhibitors at the following final concentrations; 10 µM rotenone (R), 3 µM oligomycin (O), and 100 nM antimycin A (A) for 24h.

(A) Western blot image of mitochondrial fusion protein OPA1.

(B-C) Western blot image of the inner mitochondrial membrane proteases OMA1 (B) and YME1L (C). Actin was used as an internal loading control. Images were taken by ChemiDoc imager and analyzed using Image Lab V 6.1 software.

**Supplementary Table S1: The list of differentially expressed genes (DEGs) in shPA200 versus shCTRL.** The table includes statistically up- and downregulated genes upon PA200 silencing. The threshold value for differential gene expression was set at ±1.3. The gene list was generated in StrandNGS software (www.strand-ngs.com) based on Illumina NextSeq500-derived RNA-Seq data.

**Supplementary Table S2. Gene ontology (GO) for genes activated or repressed by PA200.** The contribution of PA200-dependent genes (listed in Supplementary Table S1) to biological processes was tested with PANTHER Overrepresentation Fisher test. GOs for genes, which are up- and downregulated in response to PA200 silencing, are presented in two separate sheets.

**Supplementary Methods**

**Tetramethylrhodamine ethyl ester (TMRE) staining**

Cells were seeded in 6-well plates at the density 10^5^ cells per well. On the following day, Cells were treated for 24 hours with vehicle (DMSO, 1% v/v) or 3 μM oligomycin. After 24h, the media was removed, cells were rinsed with serum-free DMEM and were incubated with 100 nM

TMRE (Abcam) in DMEM at 5% CO_2_, 37 °C for 10min. Following incubation cells were harvested by Trypsin-EDTA, washed twice with sterile 1x PBS and resuspended in 200 µl 1x PBS complemented with 1.0% (v/v) FBS. Cells were filtered through a 41 µm Nylon Net Filter (Merck Millipore) prior to the FACS measurement. Data acquisition was performed using a FACS Aria III flow cytometer (BD Biosciences). Data were analyzed with FlowJo software.

**Reactive oxygen species (ROS) measurement**

Cells were seeded in 6-well plates at the density 10^5^ cells per well. On the following day, cells were treated for 24 hours with vehicle (DMSO, 1% v/v) or 3 μM oligomycin. After 24h, the media was removed, cells were rinsed and incubated with 1 μM Carboxy-H2DCFDA (ThermoFisher) in DMEM with reduced FBS (2%) for 30 min at 5% CO_2_, 37 ºC. Cells were harvested by Trypsin-EDTA, rinsed with 1X PBS and resuspended in 1X PBS complemented with 1% (v/v) FBS.

Cells were filtered through a 41 µm Nylon Net Filter (Merck Millipore) prior to the FACS measurement. Data acquisition was performed using a FACS Aria III flow cytometer (BD Biosciences). Data were analyzed with FlowJo software.
