# Supplementary figures and images for: Cells Lacking PA200 Adapt to Mitochondrial Dysfunction by Enhancing Glycolysis via Distinct Opa1 Processing
Source: Int J Mol Sci. 2021 Feb 5;22(4):1629. doi: 10.3390/ijms22041629 (PMC7914502; doi:10.3390/ijms22041629)

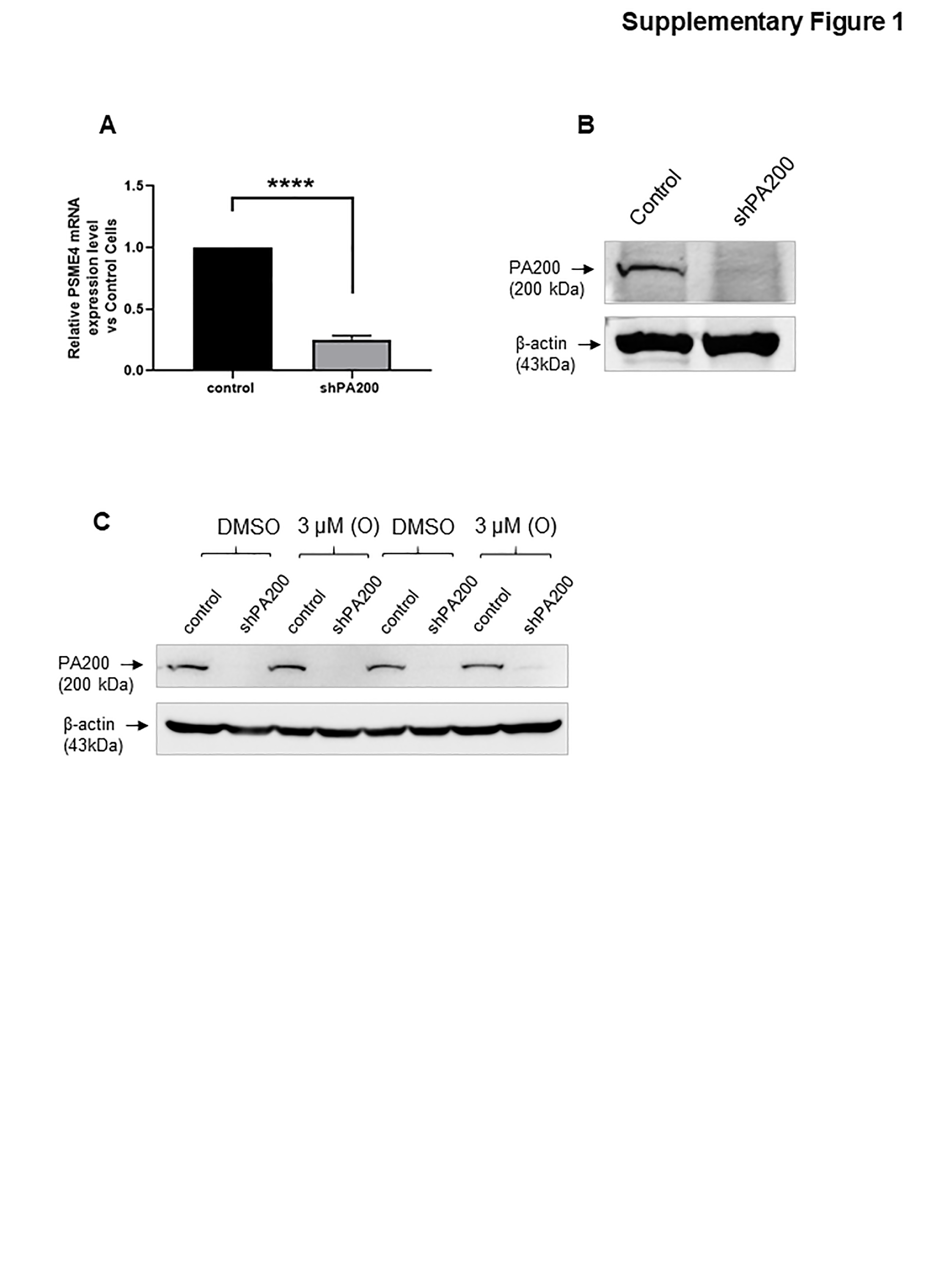

Supplement: Supplementary file 1 [file ijms-22-01629-s001.zip › supplementary/SF1.TIF]

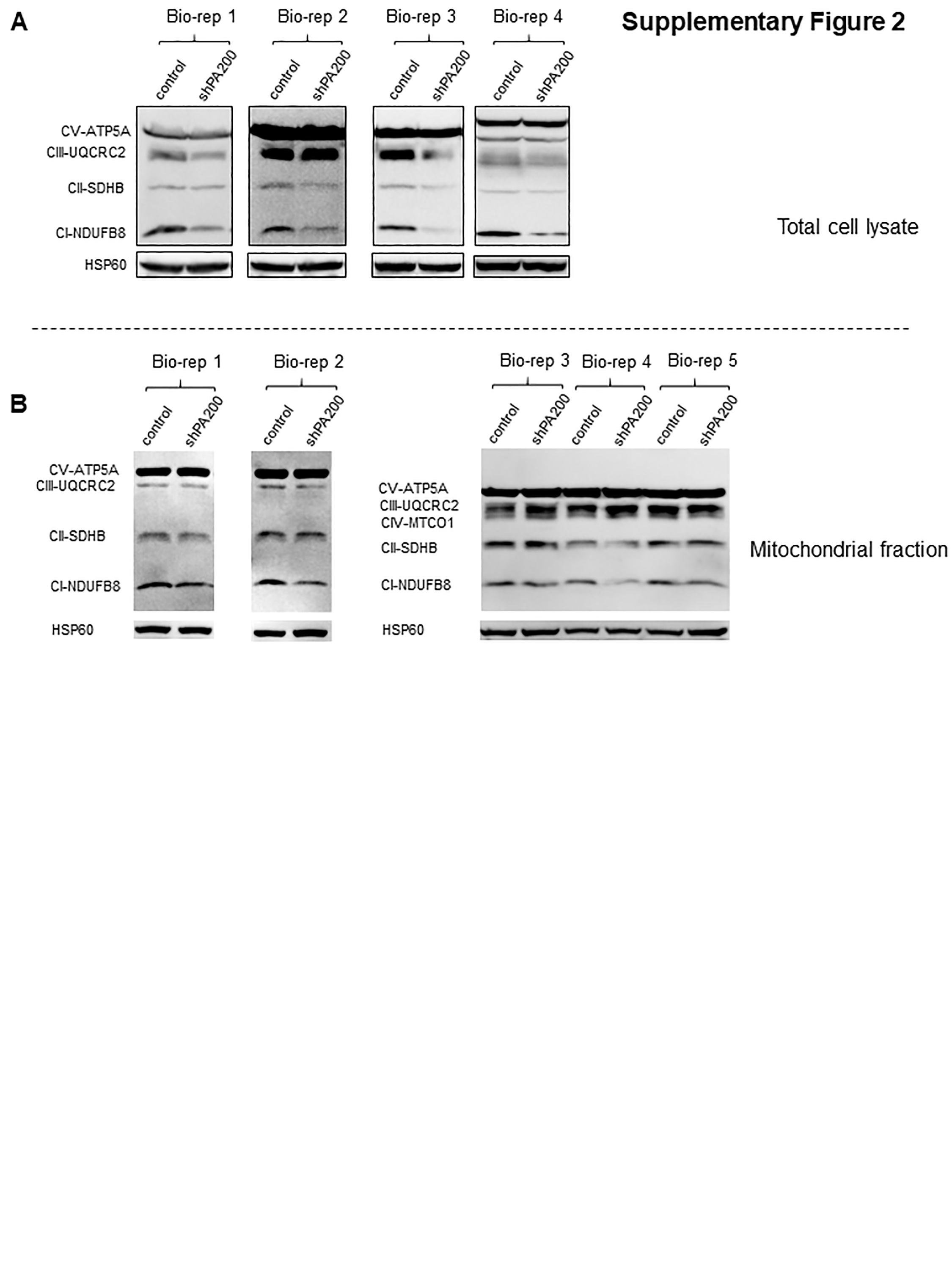

Supplement: Supplementary file 1 [file ijms-22-01629-s001.zip › supplementary/SF2.TIF]

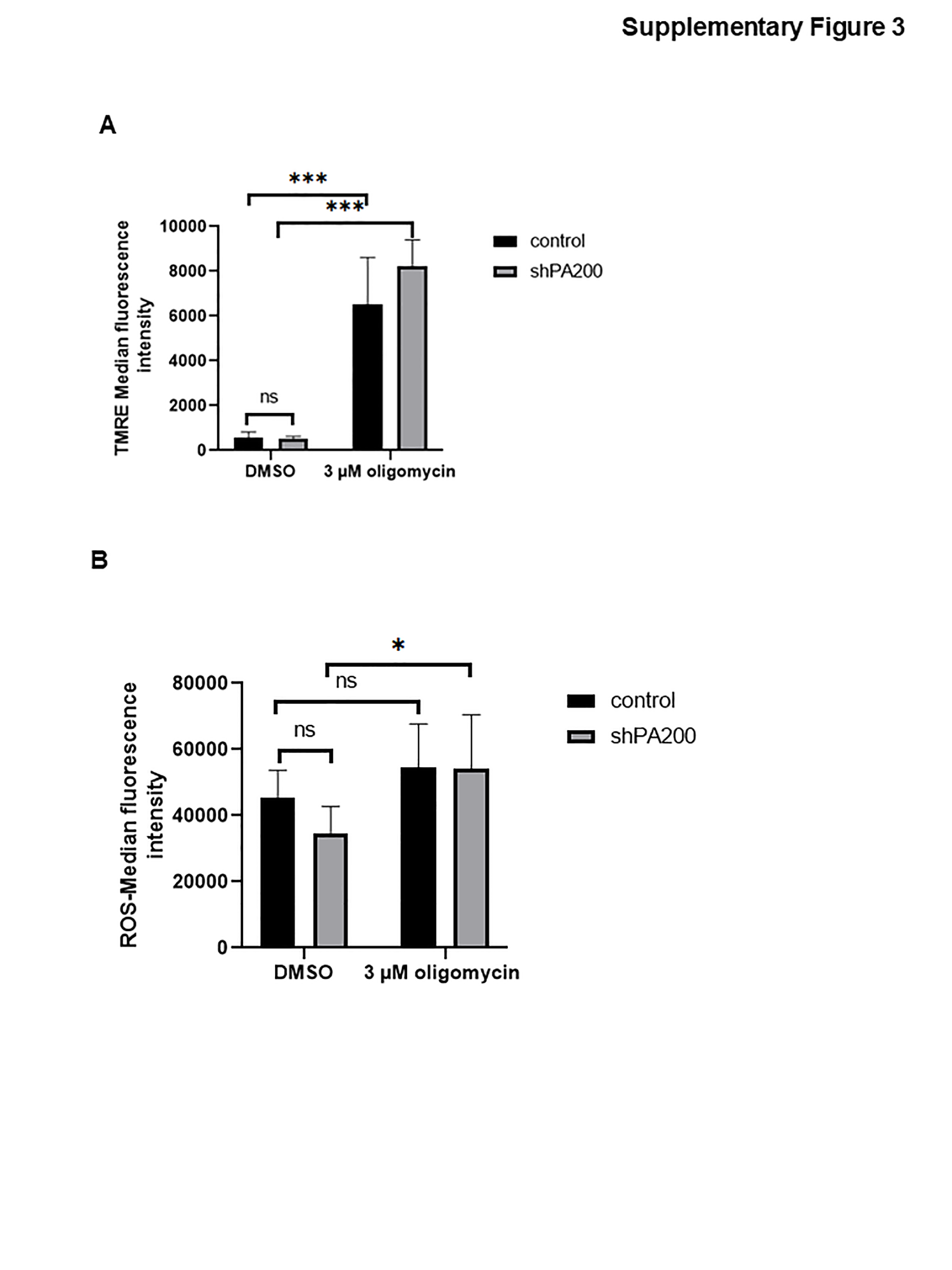

Supplement: Supplementary file 1 [file ijms-22-01629-s001.zip › supplementary/SF3.TIF]

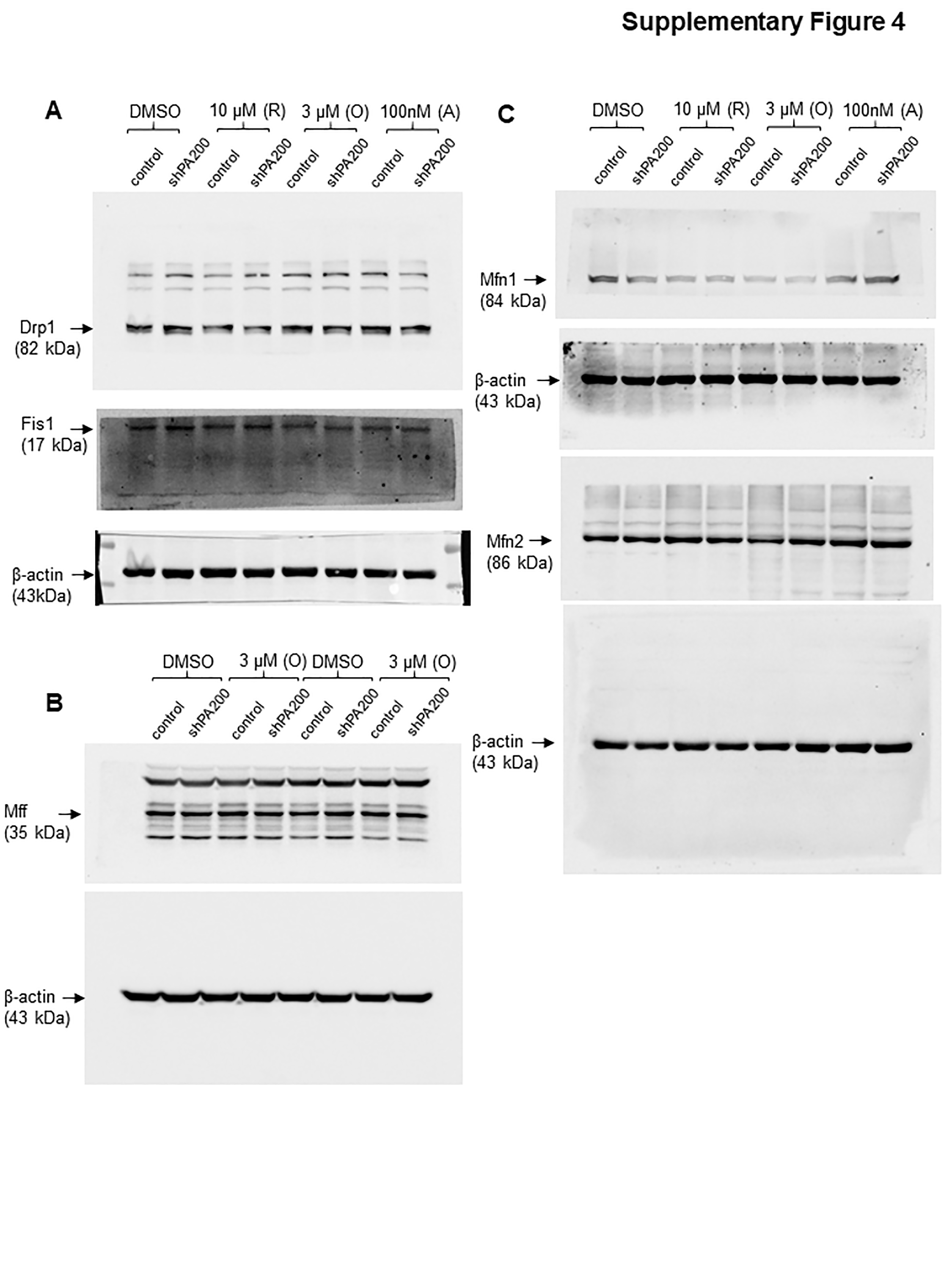

Supplement: Supplementary file 1 [file ijms-22-01629-s001.zip › supplementary/SF4.TIF]

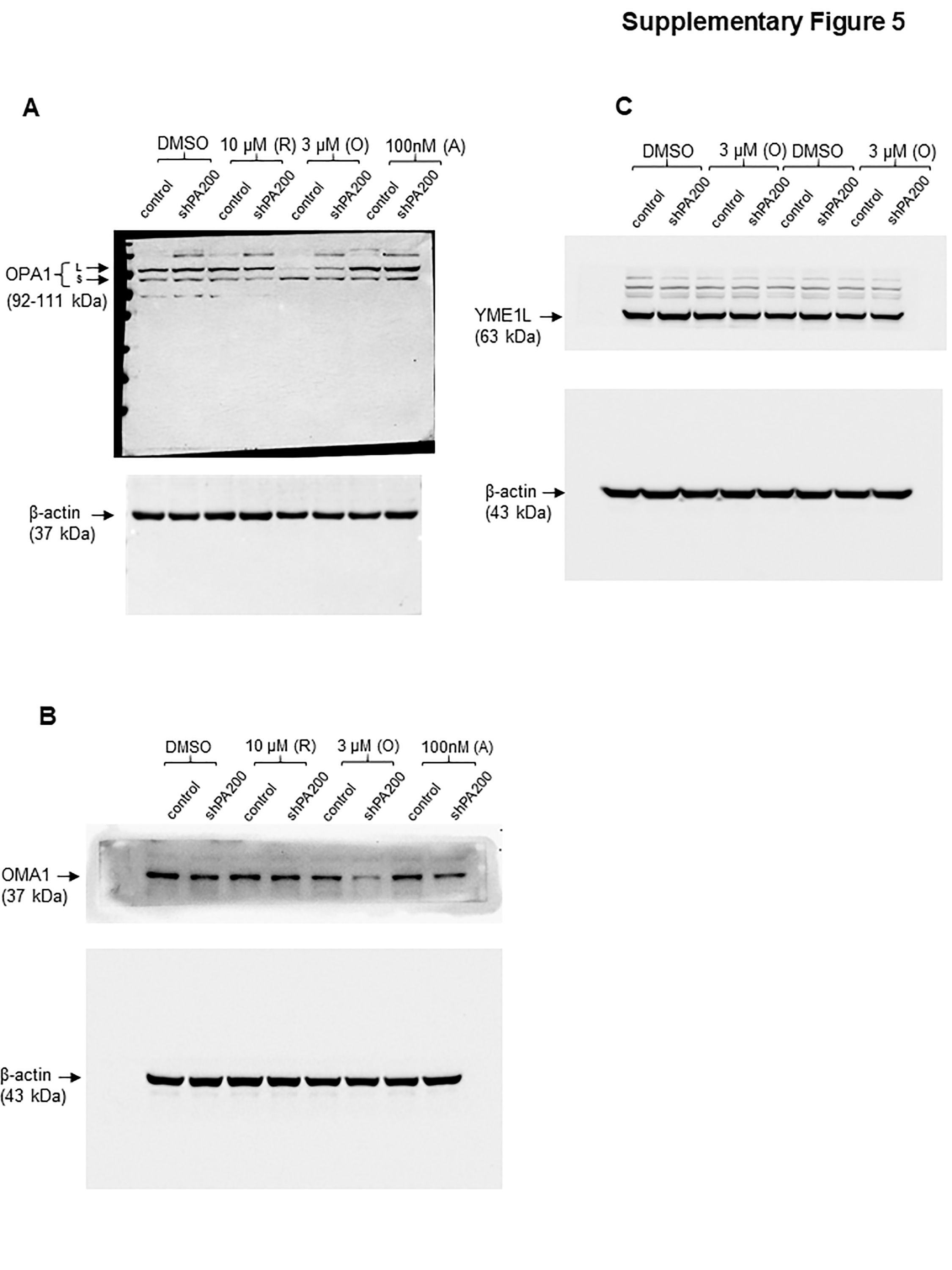

Supplement: Supplementary file 1 [file ijms-22-01629-s001.zip › supplementary/SF5.TIF]
